# Supplementary material for: Research on epilepsy detection and recognition based on the combination of time frequency transform and deep learning model
Source: PLoS One. 2026 Mar 20;21(3):e0336764. doi: 10.1371/journal.pone.0336764 (PMC13004368; doi:10.1371/journal.pone.0336764)
Supplement: S1 Table — (PDF) [file pone.0336764.s001.pdf]

No.	Research Title	Research Objectives	Methodology	Datasets Used	Key Contributions	Identified Limitations
[1]	vEpiNet: A multimodal interictal epileptiform discharge detection method based on video and electroencephalogram data	Develop a multimodal detection method for interictal epileptiform discharges using video and EEG data	Construct the vEpiNet model to fuse video and EEG data for multimodal feature extraction and detection	Specific dataset details (sample size, patient information, etc.) not explicitly mentioned	Propose a video-EEG multimodal fusion detection framework, expanding the data source dimension for epileptiform discharge detection	Not explicitly mentioned; failure to clarify the detailed fusion mechanism between video data and EEG data
[2]	EEG based BCI for Autonomous Control: A Review	Systematically review the research status, technical paths, and application prospects of EEG-based Brain-Computer Interfaces (BCI) in autonomous control	Literature review method to sort out the technical evolution, core algorithms, application scenarios, and research gaps in this field	No specific experimental datasets; based on existing research literature	Establish a research framework for BCI autonomous control, providing direction for subsequent research; summarize key challenges of EEG signal processing in autonomous control	Failure to focus on epileptic detection scenarios, with weak direct relevance to epileptic EEG analysis
[3]	An autoencoder model based on 1D convolutional neural network for epileptic seizure detection	Construct an autoencoder model based on 1D convolutional neural network to achieve efficient detection of epileptic seizures	Design a 1D-CNN autoencoder architecture: the encoder extracts temporal features of EEG signals, and the decoder reconstructs signals to distinguish epileptic/normal samples	Specific dataset details not explicitly mentioned	Propose a lightweight 1D-CNN autoencoder architecture that does not require complex time-frequency transformation, improving detection efficiency	Failure to clarify model generalization performance; failure to illustrate robustness to EEG noise
[4]	A review of EEG data analysis technology based on deep learning	Comprehensive review of the method classification, application scenarios, and development trends of deep learning-based EEG data analysis technology	Literature review method, classified and sorted by model types (CNN, RNN, Transformer, etc.) and application scenarios (epileptic detection, brain function decoding, etc.)	No specific experimental datasets; based on existing research literature	Systematically summarize the core technical paths of deep learning in EEG analysis; clarify technical hotspots and research gaps in epileptic detection	Failure to conduct in-depth special analysis on epileptic detection; failure to compare the advantages and disadvantages of different technologies
[5]	MCAN: A cross-domain hybrid self-supervised attention network for intelligent and efficient epileptic detection	Construct a cross-domain hybrid self-supervised attention network (MCAN) to address the generalization problem of epileptic detection in cross-domain scenarios	Integrate self-supervised learning and attention mechanism, design a cross-domain feature transfer module to improve model adaptability to different data distributions	Specific dataset details not explicitly mentioned	Propose a cross-domain detection framework to solve the generalization deficiency caused by "domain shift" in epileptic detection; reduce reliance on labeled data through self-supervised learning	Failure to clarify specific types of cross-domain scenarios (e.g., data from different hospitals, different devices); failure to illustrate specific target of attention mechanism
[6]	CSBrain: A Cross-scale Spatiotemporal Brain Foundation Model for EEG Decoding	Construct a cross-scale spatiotemporal brain foundation model (CSBrain) to improve the decoding ability of EEG signals (including epilepsy-related features)	Design a cross-scale spatiotemporal feature extraction module to capture multi-scale temporal dynamics and spatial distribution features of EEG signals	Specific dataset details not explicitly mentioned	Propose a foundation model architecture for EEG decoding, providing a general feature extraction platform for tasks such as epileptic detection	Failure to conduct special optimization and verification for epileptic detection; feasibility of lightweight deployment of the foundation model not clarified
[7]	A Method for Epileptic EEG Recognition Based on Semi-Supervised Deep Convolutional Channel Attention Single-Class Network	Address the scarcity of epileptic labeled data, propose a semi-supervised deep convolutional channel attention single-class network for epileptic EEG recognition	1. Preprocessing: channel selection, power frequency interference removal, sliding window segmentation; 2. Network architecture: embedding layer + wavelet decomposition (DB4) time-frequency feature extraction layer + channel attention layer + multi-level reconstruction network; 3. Training strategy: unsupervised pre-training (only normal EEG) + supervised fine-tuning (a small amount of epileptic EEG)	CHB-MIT Dataset	Realize effective application of semi-supervised learning in epileptic detection, with AUC=0.9753, sensitivity=0.9407, specificity=0.9632; channel attention mechanism enhances key feature representation	Failure to clarify model adaptability to different seizure types; failure to analyze sensitivity of wavelet decomposition parameters
[8]	Stereoelectroencephalography (SEEG) in the presurgical evaluation of epilepsy: methodology and results in 500 patients	Explore the application value of stereoelectroencephalography (SEEG) in localizing epileptogenic zones and providing precise targets for epilepsy surgery	Retrospectively analyze the clinical data of 500 epilepsy patients who received SEEG examination; standardize the SEEG implantation and signal interpretation process for presurgical evaluation	500 clinical epilepsy patients who underwent presurgical SEEG assessment	Systematically verify the efficacy of SEEG in epileptogenic zone localization; provide a reliable methodological basis for guiding epilepsy surgical treatment	The study is retrospective; lacks prospective controlled trials to further validate the long-term outcomes of surgery
[9]	Ambulatory EEG and automated seizure detection: impact on pediatric epilepsy management and quality of life	Evaluate the role of ambulatory EEG combined with automated seizure detection technology in pediatric epilepsy screening, long-term monitoring and preoperative evaluation	Deploy ambulatory EEG devices for long-term continuous monitoring of pediatric patients; apply automated detection algorithms to identify epileptic discharges; analyze the impact of detection results on treatment plan optimization	Pediatric epilepsy patients (age range not specified in the core context) receiving ambulatory EEG monitoring	Highlight the clinical value of automated detection technology in pediatric epilepsy management; confirm its positive effect on improving diagnostic efficiency and patients' quality of life	The automated algorithm's sensitivity for detecting weak epileptic discharges needs further improvement; lacks data on its application in rare types of pediatric epilepsy
[10]	Dissimilarity-based time–frequency distributions as features for epileptic EEG signal classification	Propose dissimilarity-based time-frequency distribution features for epileptic EEG signal classification	Extract dissimilarity-based time-frequency distribution features of EEG signals, and classify using traditional classification algorithms (e.g., SVM)	Specific dataset details not explicitly mentioned	Propose a novel time-frequency feature extraction method that achieves efficient classification without complex models	Feature extraction process relies on manual design; insufficient adaptability to individual differences in EEG signals
[11]	Advances in classification of EEG signals via evolving fuzzy	Improve EEG signal classification performance	Integrate evolving fuzzy classifiers with multiple HMMs	Specific dataset details not	Promote the optimization of traditional machine	High model complexity and insufficient

No.	Research Title	Research Objectives	Methodology	Datasets Used	Key Contributions	Identified Limitations
	classifiers and dependant multiple HMMs	(including epileptic detection) based on evolving fuzzy classifiers and dependent multiple Hidden Markov Models (HMMs)	to model temporal dynamic features of EEG signals	explicitly mentioned	learning models in EEG classification; improve the model's ability to capture temporal features of EEG	real-time performance; limited robustness to high-noise EEG signals
[12]	Advancing water quality assessment and prediction using machine learning models, coupled with explainable artificial intelligence (XAI) techniques	Optimize the interpretability of machine learning models for water quality assessment and prediction by combining XAI techniques (e.g., SHAP)	Adopt machine learning models for water quality prediction, and explain model decision-making process through SHAP method	Water quality-related datasets (no direct relevance to epileptic EEG)	Verify the effectiveness of XAI techniques in model interpretability, providing reference ideas for "black-box" models in epileptic detection	No direct relevance to epileptic EEG detection; technical transferability needs further verification
[13]	A novel ANN-based classification of spike-wave activity in 24-hour EEG recordings in rats using spectrograms: Spike-Wave Discharge Artificial Neural Network (SWAN)	Construct the SWAN model to classify spike-wave activity in 24-hour rat EEG based on spectrograms	Extract EEG spectrogram features and construct an Artificial Neural Network (ANN) model for classification	24-hour EEG recording data of rats	Specifically designed for animal epilepsy models, realizing accurate classification of spike-wave activity; providing technical support for basic epilepsy research	Only applicable to rat models with limited clinical transformation value; failure to consider differences among different epilepsy models
[14]	Automatic seizure detection using orthogonal matching pursuit, discrete wavelet transform, and entropy based features of EEG signals	Integrate Orthogonal Matching Pursuit (OMP), Discrete Wavelet Transform (DWT), and entropy features to realize automatic epileptic seizure detection	1. Feature extraction: OMP features + DWT time-frequency features + entropy features; 2. Classifier: Support Vector Machine (SVM)	Specific dataset details not explicitly mentioned	OMP-based method: specificity=96.58%, accuracy=97%, sensitivity=97.08%; DWT-based method: sensitivity=99.39%, accuracy=99.63%, specificity=99.72%	Cumbersome feature extraction steps and insufficient real-time performance; reliance on traditional classifiers with limited generalization
[15]	EEG-based cerebral pattern analysis for neurological disorder detection via hybrid machine and deep learning approaches	Integrate a hybrid model of Random Forest (RF) and CNN to detect neurological disorders (including epilepsy) based on EEG cerebral pattern analysis	1. Machine learning branch: RF extracts manually designed features; 2. Deep learning branch: CNN extracts image-based features; 3. Feature fusion and classification	Specific dataset details not explicitly mentioned	Address the limitations of single models, improve the comprehensiveness of neurological disorder detection; balance the interpretability of manual features and the representation ability of deep features	Failure to clarify specific performance in epileptic detection; high computational complexity of the hybrid model
[16]	Enhanced epilepsy detection using discrete wavelet transform and bandpass filtering on EEG data: integration of ART-based and LVQ models	Combine DWT, bandpass filtering, and ART-LVQ models to enhance the robustness of epileptic detection to non-stationarity and noise	1. Preprocessing: DWT denoising + bandpass filtering; 2. Classification model: integrate ART-based model and Learning Vector Quantization (LVQ) model	Specific dataset details not explicitly mentioned	Effectively reduce the interference of EEG non-stationarity and noise; improve the stability of epileptic detection	Failure to verify model adaptability to epileptic discharges in different frequency ranges; manual preprocessing steps rely on empirical parameters
[17]	Advanced epilepsy detection model using electroencephalography signals based on S-transform and ConvNeXt neural network	Construct an advanced epileptic detection model based on S-transform and ConvNeXt	1. Feature extraction: S-transform to obtain EEG time-frequency features; 2. Classification model: ConvNeXt neural network	Specific dataset details not explicitly mentioned	Excellent performance: accuracy=98.83%, specificity=97.68%, sensitivity=96.86%, Kappa coefficient=0.9551	Failure to illustrate the optimization process of S-transform parameters; insufficient lightweight degree of the ConvNeXt model
[18]	Phase spectrogram of EEG from S-transform Enhances epileptic seizure detection	Improve epileptic seizure detection effect using S-transform-based EEG phase spectrograms	1. Feature extraction: S-transform to obtain phase spectrograms; 2. Model: CNN + post-processing of channel fusion	CHB-MIT Database, Bonn Database	Verify the effectiveness of phase features: AUC-ROC increased by 6.68% on CHB-MIT dataset; sensitivity=79.59% and specificity=92.23% after channel fusion	Insufficient explanation of the physical significance of phase features; failure to verify the generality of the channel fusion strategy
[19]	Detection of epileptic seizure using EEG signals analysis based on deep learning techniques	Propose a collaborative deep learning model to realize epileptic seizure detection based on CWT/STFT time-frequency maps	Construct a dual-branch CNN model with CWT scalograms and STFT spectrograms as inputs respectively, and output classification results collaboratively	Bonn University Dataset, CHB-MIT Dataset	CWT branch: accuracy=99.57% on both Bonn/CHB-MIT datasets; STFT branch: accuracy=99.26% on Bonn dataset, 97.12% on CHB-MIT dataset	High computational cost of the dual-branch model; failure to consider adaptive adjustment of time-frequency transformation parameters
[20]	Investigating population-specific epilepsy detection from noisy EEG signals using deep-learning models	Study population-specific epileptic detection from noisy EEG signals and reveal age-related differences	Construct a deep learning model to detect noisy EEG; add age stratification analysis	Specific dataset details not explicitly mentioned	Discover age-related detection differences: significantly higher accuracy in populations under 40 years old; provide reference for population-specific detection	Failure to clarify the impact of noise types and intensities on detection performance; sample size may be insufficient to support age stratification conclusions
[21]	A wavelet-chaos methodology for analysis of EEGs and EEG subbands to detect seizure and epilepsy	Propose a wavelet-chaos methodology to analyze EEG and its subbands for epileptic seizure detection	1. Preprocessing: wavelet transform to decompose EEG subbands; 2. Feature extraction: chaos analysis (e.g., Lyapunov exponent); 3. Classification and recognition	Specific dataset details not explicitly mentioned	Pioneer the wavelet-chaos fusion analysis idea, improving the recognition of epileptic discharge features	Complex calculation of chaos features and poor real-time performance; reliance on experience for EEG subband selection
[22]	Comparison of STFT and wavelet transform methods in determining epileptic seizure activity in EEG signals for real-time application	Compare the performance of STFT and wavelet transform in real-time epileptic seizure detection	Process EEG signals using STFT and wavelet transform respectively, and evaluate classification accuracy and real-time performance	Specific dataset details not explicitly mentioned	Clarify the advantages and disadvantages of the two time-frequency transforms: wavelet transform is superior in	Failure to verify with specific classification models; lack of quantitative indicators for real-time

No.	Research Title	Research Objectives	Methodology	Datasets Used	Key Contributions	Identified Limitations
					non-stationary signal processing, while STFT has stronger real-time performance	evaluation
[23]	Wavelet transform applications in biomedical engineering	Review the applications of wavelet transform in biomedical engineering (including EEG analysis)	Literature review method to sort out the application scenarios and methods of wavelet transform in biomedical signal (EEG, ECG, etc.) processing	No specific experimental datasets; based on existing research literature	Systematically summarize the core value of wavelet transform in EEG analysis; provide theoretical support for feature extraction in epileptic detection	Failure to focus on special applications in epileptic detection; no involvement in the combination of wavelet transform and deep learning
[24]	An Attention-Based Wavelet Convolution Neural Network for Epilepsy EEG Classification	Construct an attention-based wavelet convolution neural network to improve the accuracy of epileptic EEG classification	1. Feature extraction: wavelet transform + CNN; 2. Optimization: add attention mechanism to strengthen key features	Specific dataset details not explicitly mentioned	Integrate the time-frequency extraction ability of wavelet transform and the feature selection ability of attention mechanism; improve classification accuracy	Failure to clarify the target of the attention mechanism; failure to verify model adaptability to different epilepsy types
[25]	Revised Tunable Q-Factor Wavelet Transform for EEG-Based Epileptic Seizure Detection	Improve the Tunable Q-Factor Wavelet Transform (TQWT) to optimize feature extraction for epileptic seizure detection	Improve the adaptive parameter adjustment mechanism of TQWT to extract robust time-frequency features of EEG signals	Specific dataset details not explicitly mentioned	Propose an adaptive TQWT method to improve the ability to capture features of epileptic discharges at different frequencies	Failure to compare performance differences before and after TQWT improvement; failure to illustrate the computational cost of parameter adjustment
[26]	Wavelet analysis of electroencephalogram signals	Explore the application of wavelet analysis in EEG signal processing (including epileptic feature extraction)	Process EEG signals using multiple wavelet basis functions (e.g., db4, sym8) and analyze the correlation between wavelet coefficients and epileptic discharges	Specific dataset details not explicitly mentioned	Verify the effectiveness of wavelet analysis in EEG feature extraction; provide basic method support for epileptic detection	Failure to verify performance with classification models; lack of unified standards for wavelet basis function selection
[27]	Signals and Systems	Explain signal and system theory to provide a theoretical basis for the processing of biomedical signals such as EEG	Explain basic methods from the perspective of signal analysis and transformation (Fourier transform, wavelet transform, etc.)	No specific experimental datasets; theoretical work	Provide core theoretical support for EEG signal processing; guide the selection of time-frequency transformation methods in epileptic detection	Pure theoretical work without direct experimental verification; failure to design special methods for epileptic detection
[28]	EEGNet: a compact convolutional neural network for EEG-based brain-computer interfaces	(Duplicate with [8]) Design a compact CNN model EEGNet adapted to EEG signal characteristics for BCI applications	(Consistent with [8]) Depthwise separable convolution architecture, optimizing parameters and computational efficiency	(Consistent with [8]) Specific dataset details not explicitly mentioned	(Consistent with [8]) The first compact CNN model specifically designed for EEG, widely used in epileptic detection	(Consistent with [8]) Limited ability to process long-term EEG signals; no attention mechanism
[29]	Deep learning with convolutional neural networks for EEG decoding and visualization	(Duplicate with [9]) Use CNN to decode EEG and improve interpretability through visualization	(Consistent with [9]) CNN feature extraction + heatmap visualization	(Consistent with [9]) Specific dataset details not explicitly mentioned	(Consistent with [9]) Promote the application of CNN in EEG decoding; emphasize interpretability	(Consistent with [9]) Weak clinical relevance of visualization methods
[30]	Batch Normalization: Accelerating Deep Network Training	Propose the Batch Normalization (BN) method to accelerate deep network training and improve stability	Design a BN layer to normalize network inputs and alleviate the vanishing gradient problem	Multiple general datasets (no special epileptic EEG datasets)	Provide a training optimization scheme for deep learning models in epileptic detection (e.g., CNN, Transformer); improve model convergence speed and stability	Failure to verify the effectiveness of BN on epileptic EEG data; failure to analyze the impact of BN on small-sample data
[31]	The Elements of Statistical Learning	Explain statistical learning theory to provide theoretical support for machine learning models in epileptic detection	Explain the principles and optimization methods of statistical learning algorithms (e.g., SVM, RF)	No specific experimental datasets; theoretical work	Guide the design and parameter optimization of traditional machine learning models in epileptic detection	Pure theoretical work without direct experimental verification; no involvement in the combination of deep learning and EEG
[32]	Focal Loss for Dense Object Detection	Propose Focal Loss to address the class imbalance problem	Design α and γ parameters to adjust the weight of hard-to-classify samples and optimize the loss function	General object detection datasets (no special epileptic EEG datasets)	Provide a solution for class imbalance (far more normal samples than epileptic samples) in epileptic detection	Failure to verify parameter selection on epileptic EEG data; failure to analyze the increase in computational cost
[33]	Focal loss for dense object detection	(Journal extended version of [32]) Improve Focal Loss theory and expand application scenarios	Deepen the theoretical analysis of Focal Loss and add multi-scenario experimental verification	General datasets (no special epileptic EEG datasets)	Further promote the application of Focal Loss in class imbalance tasks; provide a more mature loss function option for epileptic detection	Failure to conduct special verification on epileptic EEG data; no mention of adaptive parameter adjustment mechanism

No.	Research Title	Research Objectives	Methodology	Datasets Used	Key Contributions	Identified Limitations
[34]	Imbalanced epileptic seizure detection via focal loss and multi-scale feature fusion	Combine Focal Loss and multi-scale feature fusion to address the class imbalance problem in epileptic detection	1. Feature extraction: multi-scale CNN to extract features of different levels; 2. Loss function: Focal Loss; 3. Feature fusion strategy	Specific dataset details not explicitly mentioned	Effectively alleviate the impact of class imbalance; multi-scale features improve the detection ability of epileptic discharges of different intensities	High computational complexity of the feature fusion strategy; failure to verify the sensitivity of Focal Loss parameters
[35]	Evaluation: from precision, recall and F - measure to ROC, informedness, markedness & correlation	Systematically explain the principles and applications of model evaluation metrics (precision, recall, ROC, etc.)	Theoretically analyze the calculation methods, application scenarios, and limitations of various evaluation metrics	No specific experimental datasets; theoretical research	Provide a comprehensive evaluation metric system for epileptic detection models; guide the scientificity of result analysis	Pure theoretical research without direct experimental verification; failure to optimize metrics for the particularity of epileptic detection (e.g., high sensitivity requirements)
[36]	Cross-subject seizure detection with vision transformer and unsupervised domain adaptation	Combine Vision Transformer (ViT) and Unsupervised Domain Adaptation (UDA) to improve the generalization of cross-subject epileptic detection	1. Feature extraction: ViT to capture global temporal features; 2. Domain adaptation: adversarial network + Transfer Adaptation Module (TAM) + Discriminative Clustering Module (DCM)	CHB-MIT Dataset	Excellent performance in cross-subject scenarios: accuracy=89.20%, recall=91.05%; solve the "subject shift" problem	High computational complexity of the ViT model; cumbersome parameter adjustment of the UDA module
[37]	Deep learning based automatic seizure prediction with EEG time-frequency representation	Realize automatic epileptic seizure prediction based on S-transform time-frequency representation and Multi-channel ViT (MViT)	1. Feature extraction: S-transform to obtain time-frequency features; 2. Model: MViT to extract spatial features; 3. Optimization: K-of-N strategy	CHB-MIT Dataset<br>SH-SDU Dataset<br>DU	Seizure prediction accuracy=97.5%; lightweight design of MViT improves deployment feasibility	Failure to clarify the prediction lead time window; failure to verify the processing efficiency of long-term EEG
[38]	Single-channel seizure detection with clinical confirmation of seizure locations using CHB-MIT dataset	Realize single-channel epileptic detection and combine with clinical seizure location confirmation based on the CHB-MIT dataset	Construct multi-version detectors (single-channel, 18-channel, 4-channel) and integrate clinical spatial features	CHB-MIT Dataset	Single-channel detector recall=97.05-100%; combining clinical seizure locations improves practicality	Only verified based on the CHB-MIT dataset, generalization needs to be expanded; single-channel data loses part of spatial information
[39]	Automatic seizure detection by convolutional neural networks with computational complexity analysis	Construct an 8-layer CNN to realize automatic epileptic detection and analyze computational complexity	8-layer CNN architecture (no manual feature required); quantitative analysis of computational complexity (number of parameters, inference time)	CHB-MIT Dataset	Accuracy=96.99%, recall=97.06%; provide computational complexity analysis to support clinical deployment	Insufficient model depth, limited ability to capture complex epileptic discharge features; failure to verify robustness to noise
