# Supplementary material for: Research on epilepsy detection and recognition based on the combination of time frequency transform and deep learning model
Source: PLoS One. 2026 Mar 20;21(3):e0336764. doi: 10.1371/journal.pone.0336764 (PMC13004368; doi:10.1371/journal.pone.0336764)
Supplement: Attachment — Submitted filename: Response to Reviewers.docx [file pone.0336764.s003.docx]

Dear Reviewers,

### **We would like to express our sincere gratitude to both of you for your careful review, constructive comments, and valuable suggestions on our manuscript. These insights have greatly helped us improve the quality, rigor, and completeness of our work. In response to the journal's review requirements and your comments, we have conducted a comprehensive revision of the manuscript, focusing on strengthening the research background, optimizing the methodological presentation, supplementing experimental details, and enhancing the clinical significance discussion. All comments have been addressed point by point, and the corresponding revisions have been made in the manuscript.**

### ****Responses to Reviewer #1****

First, we would like to thank you for your positive evaluation of our work. Your recognition encourages us greatly. We have carefully addressed all the issues you raised, and the revisions are as follows:

**Comment 1:** The first paragraph of the introduction does not contain any supporting citations. Relevant citations should be included to strengthen the context and background.

**Response:** Thank you for your insightful comment. We have revised and supplemented relevant references as supporting citations in the introduction. The specific revisions are located in lines 41-49 and 52-69 of the manuscript.

**Comment 2:** The contributions listed at the end of the introduction should be more concise, clearly emphasizing the innovative methods of the research and their potential impact on the field.

**Response:** We appreciate your suggestion. We have abridged the content at the end of the introduction, with a focus on highlighting the innovative methods of our research and their potential implications for the field. The revised content is in lines 84-98 of the manuscript.

**Comment 3:** A comprehensive summary table of relevant literature should be provided, outlining key elements such as research title, objectives, methodological approaches, datasets used, main contributions, and identified limitations.

**Response:** As suggested, we have prepared a comprehensive summary table of relevant literature, namely S1 Table: Comprehensive Summary Table of Relevant Literature. The detailed content is included in the supporting file S1_Table.pdf.

**Comment 4:** At the beginning of the Design and Implementation section, a brief summary paragraph outlining the methodology should be added, followed by a block diagram (Figure 1) illustrating the workflow.

**Response:** We have made revisions in accordance with your comment. A brief summary paragraph outlining the methodology has been added at the beginning of the Design and Implementation section, accompanied by Figure 1: Overall Workflow Diagram of the Automatic Epilepsy Detection System. The specific content is located in lines 174-193 on pages 8-9 of the manuscript.

**Comment 5:** The number of features extracted per patient should be clearly specified to clarify the input dimension of the model.

**Response:** Thank you for pointing this out. We have clearly indicated the number of features extracted per patient. The relevant information is presented in the Dataset Details section of Table 1: Experimental Data Overview Table, which is located in lines 228-230 on page 11 of the manuscript.

**Comment 6:** More detailed information about the dataset is needed, including the total number of patients, data distribution, demographic characteristics, and the preprocessing steps applied.

**Response:** We have supplemented detailed information about the dataset as required, including the total number of patients, data distribution, demographic characteristics, and applied preprocessing steps. This information is provided in Table 1: Experimental Data Overview Table, lines 228-230 on page 10 of the manuscript.

**Comment 7:** For proper validation of the model, an external validation step should be performed to evaluate the robustness and generalizability of the proposed method.

**Response:** We sincerely apologize for not being able to perform the external validation as you suggested. Due to the experimental conditions and the time required for data preparation, we only conducted the subject-independent validation experiment. The details of this experiment are described in lines 439-441 on page 23 of the manuscript. We fully recognize the importance of external validation for the overall experiment. If we have the opportunity to conduct external validation in the future, we will promptly perform the experiment and publish the results of the external validation.

**Comment 8:** Further analysis is needed to explain how Continuous Wavelet Transform (CWT) improves model performance and why it is superior to other feature extraction techniques.

**Response:** We have conducted further analysis and explanation regarding how CWT enhances model performance and why it outperforms other feature extraction techniques. The detailed content is presented in lines 270-303 on pages 13-14 of the manuscript.

**Comment 9:** A comparative discussion with relevant studies should be included to highlight similarities, differences, and performance advantages.

**Response:** As recommended, we have added a comparative discussion with relevant studies. The specific content is located in lines 502-510 on page 27 of the manuscript.

### ****Responses to Reviewer #2****

First, we would like to thank you for your positive evaluation of our work. Your recognition encourages us greatly. We have carefully addressed all the issues you raised, and the revisions are as follows:

**Comment 1:** The abstract and introduction state that "the accuracy of the CWT+EEGNet combination reaches 100%". However, Table 1 clearly reports the accuracy of this combination as 98.29%. This is a significant inconsistency. The 100% figure seems to refer to its precision, not accuracy.

**Response:** Thank you for pointing out this inconsistency. We have revised the content of the abstract and corrected the error where the experimental results in the abstract were inconsistent with those in the main text. The specific revisions are located in lines 17-34 of the manuscript.

**Comment 2:** The manuscript does not specify the degree of data splitting for the 5-fold cross-validation. For the results to be clinically meaningful and to avoid data leakage, the model must be evaluated based on patient data not seen during training (patient-independent or subject-independent validation). If segments from the same patient appear in both training and validation folds, the performance metrics will be optimistically biased and will not represent real-world generalization.

**Response:** We have added the description of the data splitting degree for 5-fold cross-validation and conducted subject-independent validation as you suggested. The introduction to the data splitting degree of 5-fold cross-validation is presented in lines 215-226 on page 10, and the details of the subject-independent validation experiment are in lines 439-441 on page 23 of the manuscript.

**Comment 3:** The results in Tables 1 and 2 are presented as single values without any variance measured. Reporting only the average accuracy, precision, and recall of the 5-fold cross-validation is insufficient. The standard deviation across folds is crucial for assessing the stability and reliability of the model.

**Response:** We have added the standard deviation values and re-summarized the experimental results as required. The updated results are presented in Tables 2-5 on pages 21-24, and the re-summary of the results is provided in lines 442-463 on pages 23-24 of the manuscript.

**Comment 4:** Please briefly explain the rationale for or references to the selection of key parameters, such as the (3, 16) kernel in EEGNet, the focal loss parameters (α=0.6, γ=2.0), and the specific scale range used in Continuous Wavelet Transform (CWT). This enhances reproducibility and methodological rigor.

**Response:** We have provided explanations for the selection of key parameters as requested. The relevant explanations are located in lines 338-345 on page 16 and lines 385-390 on page 18 of the manuscript.

**Comment 5:** The excellent results present a classic and clinically important trade-off: CWT+ShallowConvNet achieves perfect recall (no missed seizures), while CWT+EEGNet achieves perfect precision (no false alarms). Please expand the discussion to explore the clinical significance of this trade-off.

**Response:** We have expanded the discussion to explore the clinical significance of the aforementioned trade-off. The detailed discussion is presented in lines 511-572 on pages 27-30 of the manuscript.

**Comment 6:** A direct comparison of your best results with other state-of-the-art methods on the same CHB-MIT dataset, in a dedicated paragraph (or table), would better illustrate the significance of your contribution.

**Response:** As suggested, we have directly compared our best results with other state-of-the-art methods on the CHB-MIT dataset in a dedicated table. The specific content is located in lines 502-510on page 27 of the manuscript.

**Comment 7:** The current Figures 3 and 4 (confusion matrices) are placeholders. Please replace them with clear, legible, and correctly labeled versions.

**Response:** We have replaced the confusion matrices as required to ensure they are clearly displayed. The updated confusion matrices are presented in Figures 4-5 on pages 21-22 of the manuscript.

**Comment 8:** Correct reference errors in the text (e.g., [Error! Reference source not found]).

**Response:** We have carefully checked all the references in the text and corrected the reference errors as you indicated.

**Comment 9:** Please carefully check the Butterworth filter transfer function (Equation 1) for possible typos in the denominator...

**Response:** We have carefully checked Equation 1 (Butterworth filter transfer function) and corrected the typo in the denominator. The revised equation is presented in lines 326-335 on page 16 of the manuscript.

Once again, we would like to thank both reviewers for their time and valuable comments. We believe that these revisions have significantly improved the quality of our manuscript. We hope that the revised version meets the publication requirements of the journal. Please feel free to contact us if you have any further questions.

Sincerely,

Canhui Wang

School of Electrical and Information Engineering, Yunnan Minzu University

E-mail：yan.li@ymu.edu.cn
