# Supplementary material for: Research on epilepsy detection and recognition based on the combination of time frequency transform and deep learning model
Source: PLoS One. 2026 Mar 20;21(3):e0336764. doi: 10.1371/journal.pone.0336764 (PMC13004368; doi:10.1371/journal.pone.0336764)
Supplement: Attachment — Submitted filename: Response_to_Reviewers_auresp_2.docx [file pone.0336764.s004.docx]

Response to Reviewers

Manuscript ID: PONE-D-25-58659R1

Title: Research on Epilepsy Detection and Recognition Based on the Combination of Time Frequency Transform and Deep Learning Model

Dear Editor Ibrahim Sadek, Ph.D. and Reviewers,

We would like to express our sincere gratitude to you and the reviewers for your constructive comments and valuable suggestions, which have greatly helped us improve the quality and completeness of our manuscript. We have carefully addressed all the comments raised by the reviewers, and the detailed revisions are presented point-by-point as follows.

**Response to Reviewer #1**

**Comment:** Comparison between the obtained results and the relevant studies should be added to the discussion section to highlight the significancy of the proposed study.

**Response:** Thank you for your valuable suggestion. We have revised the Discussion section and added a comprehensive comparison between our obtained results and relevant existing studies. The revised content is located in Lines 510–517 of the manuscript, which clearly illustrates the advantages of our proposed method and highlights the scientific significance and innovation of this research.

**Response to Reviewer #2**

**Comment 1:** In the submitted manuscript, Table 5 (Comparison with State-of-the-Art) appears incomplete and garbled, containing repeated columns and numbers with unclear labeling. This table is critical for positioning your research contribution.

**Response:** We apologize for the formatting error in the original table. We have thoroughly revised the table, which is now labeled as Table 6: Performance comparison with state-of-the-art methods on CHB-MIT Dataset. The revised table is complete, with clear column labels and no redundant content, and is located in Lines 518–652 of the manuscript.

**Comment 2:** While most references have been corrected, there are still instances in the text (e.g., on pages 12, 13, 49) containing the placeholder phrase "[Error! Reference source not found.]". This indicates a final referencing or linking issue.

Response: Thank you for pointing out this issue. We have thoroughly checked the entire manuscript and corrected all citation errors, including the placeholder phrases mentioned. All references are now correctly linked and displayed without any abnormalities.

**Comment 3:** The confusion matrices have been replaced (Figures 4-5), which is good. However, Figures 6, 7, 8 (comparison of original signals, STFT, and CWT results) in the current PDF are low-resolution or poorly labeled.

Response: We appreciate your careful review. We have replaced all unclear figures in the manuscript; the original Figures 6, 7, and 8 have been updated and expanded to Figures 6, 7, 8, and 9, with improved resolution (meeting the journal’s ≥300 dpi requirement) and clear labels, legends, and axis annotations to ensure better readability and presentation.

**Comment 4:** In the manuscript text (Page 63 of the attached PDF), Equation 1 appears as H(s)=1 1+(- 2- 2N, which is an incorrect and uninterpretable format.

**Response:** We apologize for the formatting error in Equation 1. We have retyped and corrected Equation 1 to ensure its correctness and interpretability, and we have also checked all other mathematical equations in the manuscript to avoid similar issues.

**Comment 5:** The manuscript document contains duplicated sections, where the author response text (Response to Reviewers) appears twice (at the beginning and near the end of the document). The final submission for publication should contain only the research manuscript text, excluding the response letter.

**Response:** Thank you for reminding us of this formatting issue. We have carefully checked the manuscript and deleted all duplicated sections, including the redundant author response text. The revised manuscript only contains the main research content, which fully complies with the journal’s submission requirements.

In summary, we have addressed all the comments raised by the reviewers thoroughly. We believe that the revised manuscript has been significantly improved in terms of content completeness, formatting standardization, and presentation clarity, and now meets the publication criteria of PLOS ONE. We sincerely appreciate the reviewers’ and editor’s efforts and guidance, and we look forward to your positive evaluation.

Sincerely,

Canhui Wang

School of Electrical and Information Engineering, Yunnan Minzu University

E-mail：yan.li@ymu.edu.cn
